# A Bifunctional Organosilane Additive for Dynamic pH Regulation and Interfacial Protection in Aqueous Zinc‐Ion Batteries

**DOI:** 10.1002/advs.76526

**Published:** 2026-07-11

**Authors:** Lina Pan, Haiyang Wu, Peng Huang, Boyu Yuan, Chao Lai, Yaqian Lan

**Affiliations:** ^1^ School of Chemistry and Materials Science Jiangsu Normal University Xuzhou P. R. China; ^2^ School of Physics and Electronic Engineering Jiangsu Normal University Xuzhou P. R. China; ^3^ Guangdong Provincial Key Laboratory of Carbon Dioxide Resource Utilization South China Normal University School of Chemistry Guangzhou P. R. China

**Keywords:** aqueous zinc‐ion batteries, electrolyte additives, organosilanes

## Abstract

Aqueous zinc‐ion batteries exhibit considerable potential for use in grid‐scale energy storage. However, issues such as zinc dendrite growth and the occurrence of the hydrogen evolution reaction have limited their development. To address these challenges, this study develop Ormosil, a water‐soluble organosilane additive for the electrolyte. In the electrolyte, Ormosil forms Si─O─Zn bonds with the zinc foil, thereby helping to protect the electrode from corrosion reactions. Moreover, on the electrode surface, it facilitates the formation of a solid‐electrolyte‐interphase layer, enabling rapid zinc‐ion transport kinetics. Notably, the hydrophilicity of the organosilane is enhanced by the introduction of ─NH_2_ groups. Furthermore, the ─NH_2_ groups can bind to zinc ions and become part of their solvation shell; this incorporation helps suppress the hydrogen evolution reaction. Consequently, the single additive Ormosil provides both electrode protection and solvent restructuring. The cell using Ormosil additive exhibited excellent cycling stability (10 mA cm^−2^, 1000 h), a high average coulombic efficiency (CE = 99.74%), and a full cell with NVO cathode demonstrated high capacity retention (76.60%), superior to ZnSO_4_ (51.08%).

## Graphical abstract

A novel multifunctional additive for aqueous zinc‐ion battery electrolytes can simultaneously dynamically adjust the pH in microzones, regulate the solvation structure of Zn^2+^, protect the interface, and form a stable solid‐state SEI film. This approach enables a dendrite‐free zinc anode and demonstrates excellent cycling stability and cycle life, offering a new approach for high‐performance aqueous zinc‐ion batteries.

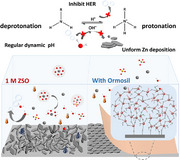

## 1 Introduction

Aqueous zinc‐ion batteries (AZIBs) are potential new energy materials because of their low cost, environmentally friendly, safe, low redox potential (0.76 V vs. standard hydrogen electrode) and high theoretical specific capacity (820 mAh g^−1^) [1, 2]. However, during cycling, zinc dendrites tend to form on the zinc anode surface, and water triggers the hydrogen evolution reaction (HER), which reduces the battery's coulombic efficiency and cycle life [3, 4, 5]. In particular, the growth of zinc dendrites is triggered by the non‐uniform electric field on the zinc surface and irreversible 2D diffusion occurring on the zinc anode surface. In the electrochemical environment, water molecules undergo weak dissociation to produce H_3_O^+^ [6]. Driven by the electric field, H_3_O^+^ migrates to the zinc anode surface, where it gains electrons and is reduced to form adsorbed hydrogen species (H), which are ultimately released as H_2_.

In recent years, researchers have increasingly recognized that zinc deposition and stripping processes are accompanied by significant local pH fluctuations: OH^−^ enrichment at the interface (pH increase) during deposition, and H^+^ release (pH decrease) during stripping [7, 8]. These dynamic pH changes not only affect the solvation structure and transport behavior of Zn^2+^ but are also intrinsically linked to dendrite growth; alkaline environments tend to induce byproduct formation, while acidic environments exacerbate hydrogen evolution corrosion. Therefore, developing a method capable of in situ dynamic regulation of pH and ion distribution in micro‐regions at the interface through the electrochemical surface oscillation effect offers a new approach to overcoming the stability bottleneck of zinc anodes. Plinio Innocenzi controlled the protonation and deprotonation states of the amino group's −NH_2_ by adjusting the pH; thus, under acidic conditions, it exists as −NH_3_^+^, and under basic conditions, it exists as −NH_2_ [9]. Therefore, by adding electrolyte additives containing −NH_2_ functional groups, it is possible to modulate the pH changes that occur during the zinc deposition and stripping process, thereby adjusting the interfacial microenvironment to suppress the adverse effects of H_2_O‐related side reactions. Second, due to their excellent stability, polysilanes are widely used for corrosion protection. Zhang et al. added VTMS to the lithium‐ion electrolyte to provide protection and flame retardancy, significantly improving the battery's cycle performance [10]. Polysilanes can adhere to the zinc surface by forming Si─O─Zn bonds, thereby creating a durable interface [11]. At the same time, they can form a uniform nanoscale structure, promoting uniform zinc plating and stripping. Therefore, silanes offer significant advantages in protecting the zinc anode in AZIBs.

**FIGURE 1:**
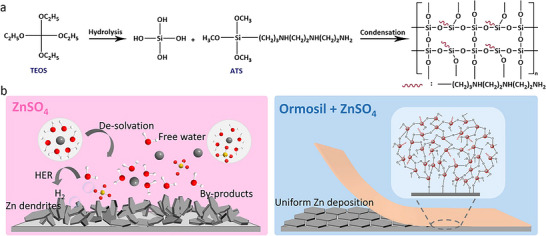
(a) Schematic of Ormosil synthesis. (b) Schematic of the mechanism through which Ormosil influences zinc deposition.

Inspired by this, this study developed a water‐soluble bifunctional organosilane additive (Ormosil) through the cross‐linking of tetraethyl orthosilicate (TEOS) and 3‐[2‐(2‐Aminoethylamino)ethylamino]propyltrimethoxysilane (ATS) (Figure 1a). The organic ─NH_2_ groups in ATS modulate the interfacial microenvironment through protonation/deprotonation processes. When dendrite growth begins, local pH changes trigger an increase in ─NH_2_ concentration, thereby electrostatically repelling Zn^2+^ aggregates. This enables real‐time regulation of dendrite growth during its early stages (at the nucleation microdomain scale). Furthermore, Ormosil forms Si─O─Zn bonds with the zinc foil, ensuring durable adhesion between the zinc foil and the Ormosil layer. This results in the formation of a solid‐electrolyte interface (SEI) layer on the electrode surface, isolating the zinc anode from direct contact with the electrolyte and inhibits the chemical corrosion of the zinc foil by water (Figure 1b). Batteries utilizing Ormosil‐modified electrolyte and zinc anodes demonstrated excellent electrochemical stability and cycle life (10 mA cm^−2^, 1000 h; 1 mA cm^−2^, 2500 h), a high average coulombic efficiency (99.74%), and a high capacity retention rate (76.60%).

## 2 Results and Discussion

### 2.1 Structure and Characterization of Electrolytes

Because ATS is nearly insoluble in water, it can be decomposed or hydrolysed to form precipitates in aqueous solutions. In contrast, ethyl orthosilicate hydrolyses in water to form orthosilicic acid (Si(OH)_4_) [12]. Therefore, Ormosil is obtained by cross‐linking orthosilicic acid, as the backbone, with ATS. The resulting Ormosil is water‐soluble. The electronic image of Ormosil is shown in Figure S1. Based on literature data and preliminary screening experiments, the effects of Ormosil concentrations of 0, 0.05, 0.1, 0.2, 0.4, 0.5, and 1 g L^−1^ were tested. With 0.05 g L^−1^ Ormosil, the cycling performance of the Zn||Zn cell did not improve significantly; with 1 g L^−1^ Ormosil, voltage polarization increased markedly (Figure S2). Therefore, in this study, control electrolytes were prepared using Ormosil at concentrations of 0.1, 0.2, 0.4 and 0.5 g L^−1^ to determine the optimal concentration of the additive [13, 14]. The cost of the electrolyte is provided in Table S3.

**FIGURE 2:**
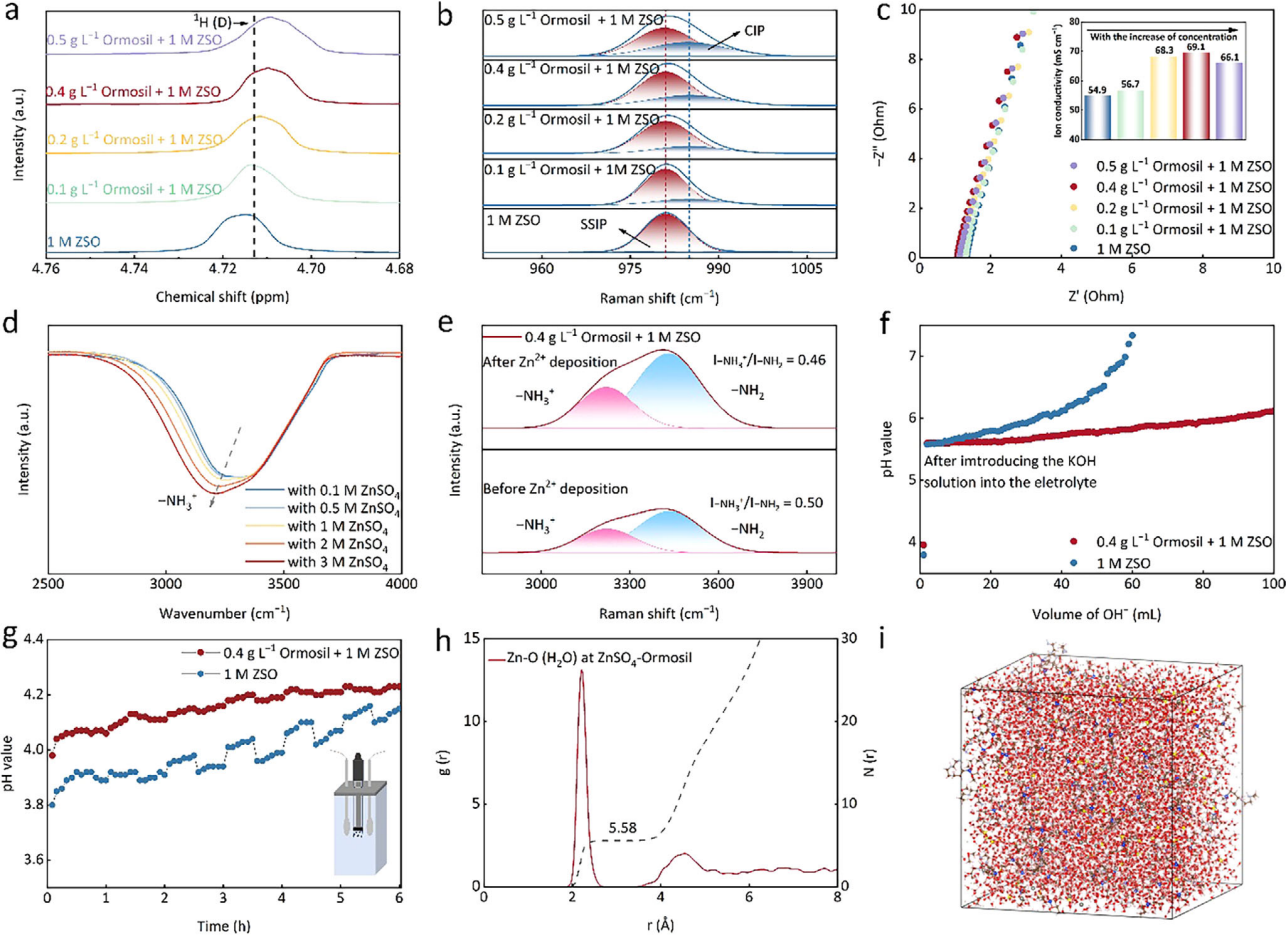
Schematic of the mechanism of action and the physicochemical properties of the electrolyte. Comparison of (a) ^1^H NMR spectra, (b) Raman spectra and (c) conductivity of electrolytes with different Ormosil concentrations. (d) The ATR‐FTIR spectrum of 0.4 g L^−1^ Ormosil in different concentrations ZnSO_4_ electrolytes. (e) Raman spectra of 0.4 g L^−1^ Ormosil + 1 M ZSO electrolyte before and after deposition. (f) Comparison of buffering capacity. (g) In situ pH values. (h) Radial distribution function of Zn─O (H_2_O) in 1 M ZSO + Ormosil electrolyte. (i) Molecular dynamics image of 1 M ZSO + Ormosil electrolyte.

The nuclear magnetic resonance (NMR) results indicate that as Ormosil concentration increases, the ^1^H peaks gradually move to a higher field. This shift is due to changes in the Zn^2+^ solvation structure caused by Ormosil addition, which replaces some H_2_O molecules and enters the Zn^2+^ primary solvation shell (Figure 2a). The effects of the electrolyte on zinc corrosion depend on factors like pH [15, 16]. The pH of 1 M ZnSO_4_ (1 M ZSO) electrolyte is approximately 3.8, indicating an acidic environment. The pH increased as the concentration of Ormosil increased, reaching 4.0 at 0.5 g L^−1^ Ormosil (Figure S3). This indicates that the ─NH_2_ groups in Ormosil provide appropriate alkalinity, enabling Ormosil to inhibit corrosion by raising the electrolyte pH to provide a weakly acidic environment [17]. To quantify the effect of Ormosil on the electrolyte's wettability, contact angles were measured. The measurement revealed that the Zn anode in 1 M ZSO has a higher contact angle of 103.3° owing to its hydrophobicity. When Ormosil was added at concentrations of 0.1, 0.2, 0.4 and 0.5 g L^−1^, the contact angle decreased to 97.6°, 93.2°, 89° and 73.9°, respectively, attributed to the favourable affinity between Ormosil and metallic zinc (Figure S4). The Raman spectra in the ν−SO_4_^2−^ band (970−990 cm^−1^) were obtained for the different electrolytes. With increasing Ormosil concentration, the ν−SO_4_^2−^ peak gradually shifted to a higher wavenumber (from 980.52 to 981.55 cm^−1^; Figure 2b). According to the electron transfer mechanism, the ν−SO_4_^2−^ bond centred at 981−985 cm^−1^ was resolved into signals of two major species: the solvent‐shared ion pair (SSIP, [Zn^2+^(H_2_O)_6_SO_4_^2−^]) and the contact ion pair (CIP, [Zn^2+^(H_2_O)_5_OSO_3_^2−^]) (Figure S5) [18, 19]. The results show that in more concentrated solutions, the Zn^2+^ coordination with the solvent molecules is suppressed, which results in desolvation of the typical [Zn(H_2_O)_6_^2+^] complex [20, 21]. In contrast, the electrolyte modified by Ormosil addition promotes SO_4_^2−^ migration into the interior of the Zn^2+^ solvation shell, resulting in a higher CIP ratio. These results suggest that the ─NH_2_ groups of Ormosil actively capture Zn^2+^, leading to locally high Zn^2+^ concentrations. The ionic conductivity of an electrolyte is a key indicator of its ability to transport ions. Ormosil additives enhance conductivity, thereby improving ion transport efficiency. The ionic conductivity of the electrolyte gradually increased from 54.9 mS cm^−2^ (1 M ZSO) to 69.1 mS cm^−2^ (0.4 g L^−1^ Ormosil + 1 M ZSO) with increasing Ormosil additive concentration. When the Ormosil concentration reached 0.5 g L^−1^, the ionic conductivity may decrease due to increased viscosity (Figure 2c) [22]. In addition, the attenuated total reflection Fourier transform in infrared (ATR‐FTIR) spectroscopy of 0.4 g L^−1^ Ormosil in ZnSO_4_ solutions of varying concentrations was measured. It can be observed that the N–H stretching vibration shifts to the red as the ZnSO_4_ concentration increases, indicating that the characteristic peak of ─NH_3_^+^ is dominant, and that ─NH_3_^+^ repels the aggregation of Zn^2+^ (Figure 2d). To verify the dynamic proton exchange mechanism involving ─NH_2_ and ─NH_3_^+^, Raman spectroscopic analysis was conducted on the electrolyte before and after deposition. The peak analysis revealed a decrease in the peak area ratio of −NH_3_^+^ to −NH_2_ after deposition (Figure 2e), indicating that interfacial alkalization promotes the deprotonation of −NH_3_^+^, leading to the dynamic release of H^+^ to buffer the pH. This provides spectroscopic evidence for the pH regulation mechanism under non‐in situ conditions [23].

Using ATR‐FTIR spectra and scanning electron microscopy (SEM) to characterise the chemical environment and composition of the electrolyte on cycled zinc plates. The ATR‐FTIR spectrum (Figure S6) clearly shows N─H stretching (3352 cm^−1^) and bending vibration (1643 cm^−1^), C─H stretching (2817 cm^−1^), C─N stretching (1545 cm^−1^) [24, 25] as well as the Si−O−Si stretching vibration (1098 cm^−1^) [26, 27]. The data indicate that the Zn anode surface has been successfully modified with an Ormosil layer. No characteristic peaks were observed on the zinc anode surface it was cycled in 1 M ZSO; the peak near 1023 cm^−1^ is the characteristic peak of Zn−OH, a byproduct generated during the recycling process. The SEM image and the corresponding energy‐dispersive x‐ray spectroscopy (EDS) map of the zinc anode after 50 cycles show a uniform distribution of N and Si across the Zn foil surface, further confirming modification of the Zn anode surface with the Ormosil layer (Figure S7). The SEM image and EDS results for the copper foil after zinc deposition in the Zn||Cu half‐cell (Figure S8) show that Ormosil was dense on the Cu surface, whereas the Zn component was distributed in the same area, indicating interaction between Zn^2+^ and Ormosil during the deposition process. The data suggest that Zn^2+^ and Ormosil interacted with each other during the deposition process. To assess the difference in the pH buffer capacity of both electrolytes, the pH of 0.1 M KOH was continuously measured. In the 1 M ZnSO_4_, the pH reached 7.34 upon adding 60 mL of 0.1 M KOH, the pH of the Ormosil‐containing electrolyte reached only 5.83. After adding 100 mL of 0.1 M KOH, the pH of the Ormosil electrolyte was merely 6.12 (Figure 2f).

The Ormosil electrolyte exhibited superior buffering capacity compared to the pure ZnSO_4_ electrolyte, indicating that Ormosil has the potential to inhibit Zn_4_SO_4_(OH)_6_·5H_2_O (ZHS) byproducts formation during battery cycling. The ability of this additive to regulate pH at the micro‐scale was demonstrated by in situ monitoring of the electrolyte's pH during the cycling process. As shown in Figure 2g, during discharge, a slight increase in pH caused the following to occur in the solution: −NH_3_^+^ → −NH_2_ + H^+^(Deprotonation). Buffering occurs through the release of H^+^. During charging, the pH rises slightly, and the following occurs: −NH_2_ + H^+^ → −NH_3_^+^(Protonation). The interface consumes H^+^, thereby inhibiting the rise in pH. During the cycle, the protonation of −NH_2_/−NH_3_^+^ and the electrochemical processes of zinc deposition and stripping form periodic oscillations over time. This allows the hydrogen evolution reaction and corrosion to be suppressed during the deposition and stripping process by controlling the pH of the electrolyte.

After immersing Zn foil in the blank and Ormosil‐modified electrolytes at different concentrations for 7 days, the SEM images showed substantial deposition of hexagonal, plate‐like ZHS byproducts on the Zn foil immersed in 1 M ZSO (Figure S9). In contrast, when Zn was immersed in the 0.4 g L^−1^ Ormosil + 1 M ZSO electrolyte, minimal ZHS byproduct was observed. X‐ray diffraction (XRD) analysis of zinc foils immersed in both the blank and 0.4 g L^−1^ Ormosil‐containing electrolytes revealed crystalline structures on the surfaces of the foil. Foils immersed in the blank electrolyte exhibited XRD peaks at 8.1°, 16.3° and 24.5° corresponding to the characteristic peaks of Zn_4_SO_4_(OH)_6_·5H_2_O. However, immerse the zinc foil in the 0.4 g L^−1^ Ormosil electrolyte, these peaks were not observed, consistent with the SEM results (Figure S10). These test results show that the Ormosil additive has excellent corrosion resistance. To verify the stability of Ormosil, the ATR‐FTIR spectra of the electrolyte were measured before and after 100 cycles. As shown in Figure S11, the Si─O─Si vibration peak (1100 cm^−1^) and the N─H peak (3200 cm^−1^) of the Ormosil‐containing electrolyte remained intact after prolonged cycling, with peak positions and relative intensities nearly identical to those of the electrolyte before cycling. This indicates that Ormosil exhibits good cycling stability during the cycling process [28]. The reactions of siloxane compounds during long‐term cycling do not adversely affect the stability of the electrolyte or the zinc‐anode interface. Siloxanes maintain long‐term cycling stability by modulating the interfacial microenvironment.

Additionally, ATR‐FTIR spectra were tested. The ν(O−H) band was detected within the 2800−3800 cm^−1^ range. Compared with the use of pure water and 1 M ZnSO_4_, the intensity of the ν(O−H) peak was suppressed upon Ormosil addition, indicating restricted mobility of the water molecules (Figure S12) [22]. Zn^2+^ typically binds to six H_2_O molecules, forming [Zn(H_2_O)_6_]^2+^ in ZnSO_4_ electrolyte. The radial and the coordination distribution function (Figures S13 and S14) reveal that H_2_O molecules in the surrounding solvent layer are located 2 Å from Zn^2+^, with an average coordination number of water molecules per Zn^2+^ (Zn^2+^−O (H_2_O)) of approximately 5.78. In the Ormosil + ZSO system, the Ormosil molecules displace H_2_O molecules around Zn^2+^ and become incorporated into the solvation shell, thus substantially modifying the solvation structure (Figure 2h,i). Moreover, when Ormosil was added, the average coordination numbers for Zn^2+^−O (H_2_O) and Zn^2+^−O(Ormosil) were 5.58 and 2 Å, respectively, indicating the formation of a new solvation structure.

### 2.2 Depositing/Stripping Performance Evaluation

**FIGURE 3:**
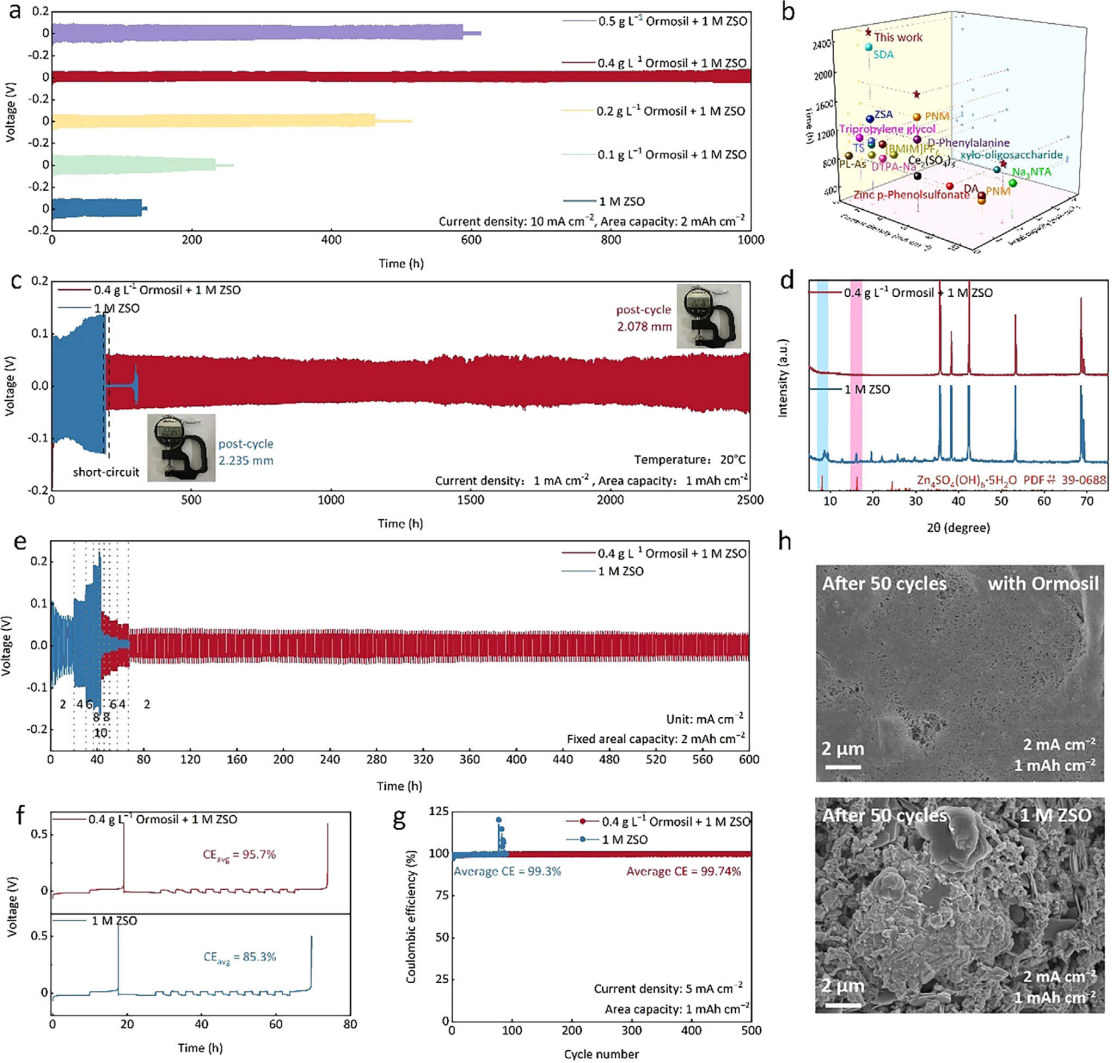
(a) Cycling tests for Zn||Zn cells in 1 M ZSO and Ormosil electrolytes were conducted at 10 mA cm^−2^ and 2 mAh cm^−2^. (b) Comparison of the Ormosil system with some previously reported electrolyte additives. (c) Cycling tests for Zn||Zn cells at 1 mA cm^−2^, 1 mAh cm^−2^. (d) XRD pattern of the Zn anode after 50 cycles. (e) Rate performance. (f) Evolution of Zn||Cu half‐cell voltage curve using the “Aurbach” method. (g) CE of Zn||Cu cell. (h) SEM images of the Cu foil surface after 50 cycles using Ormosil electrolyte and 1 M ZSO electrolyte at 2 mA cm^−2^ and 1 mAh cm^−2^.

To explore the effect of the concentration of Ormosil on the battery cycle life, Zn||Zn symmetric cells were assembled and tested to long‐cycle experiments. Figure 3a shows that the Zn||Zn cell employing the 0.1 g L^−1^ Ormosil + 1 M ZnSO_4_ electrolyte operated stably for 233 h at a current density of 10 mA cm^−2^ and a capacity of 2 mAh cm^−2^, exceeding the 128 h performance of the blank electrolyte. As the Ormosil concentration increased, the cell with 0.4 g L^−1^ Ormosil additive maintained stable operation for 1000 h under the same conditions. Furthermore, with higher additive concentrations, the polarization voltage of the Zn||Zn cell gradually decreases. It can be observed that the overpotential gradually decreases and stabilises over the first few cycles. This is because, during the initial cycles, −NH_2_ gradually establishes a protonation equilibrium on the electrode surface, forming a dynamic pH buffer layer. However, further increasing the Ormosil concentration did not improve the cycle life. The cell's cycle life was only 588 h when using an electrolyte with 0.5 g L^−1^ Ormosil additive. This is due to increased viscosity leading to higher impedance, resulting in degraded performance [29]. At 5 mA cm^−2^ and 1 mAh cm^−2^, the Zn||Zn cell employing the blank electrolyte rapidly failed owing to short‐circuiting after roughly 284 h. As expected, the Zn||Zn cell with 0.4 g L^−1^ Ormosil additive achieved extended cycling time exceeding 1800 h (Figure S15). At low current densities of 1 mA cm^−2^ and 1 mAh cm^−2^, the battery employing 0.4 g L^−1^ Ormosil additive operated stably for more than 2500 h, whereas the unmodified battery short‐circuited after cycling for less than 200 h (Figure 3c). With its Ormosil electrolyte, the Zn||Zn symmetric cell showed electrochemically competitive performance against most previously reported zinc symmetric cells that used various electrolyte additives (Figure 3b and Table S1). XRD testing was used to evaluate the role of Ormosil in stabilising the zinc anode during cycling at 5 mA cm^−2^. Figure 3d shows the Zn anode XRD patterns after 50 cycles in both electrolytes. Only the characteristic peaks of zinc metal were detected on the electrode cycled in the Ormosil‐containing electrolyte. This result confirms the efficacy of Ormosil for suppressing side reactions. The protective effect of Ormosil under high areal capacity conditions was further evaluated. At 5 mAh cm^−2^, the Zn||Zn battery cycled stably for over 400 h (Figure S16), confirming that this interfacial protection mechanism retains excellent robustness even under deep cycling conditions. Figure 3e shows the rate performance of the Zn||Zn cell. As illustrated, at a constant capacity of 2 mAh cm^−2^, encouraging rate performance was achieved across current densities from 2 to 10 mA cm^−2^. The cell employing the Ormosil additive exhibited superior rate capability, whereas that without the additive short‐circuited prematurely at a current density up to 10 mA cm^−2^.

The “Aurbach” method was used to assess the reversibility of Zn^2+^ deposition and stripping (Figure S17) [30]. The calculated CE of the Zn||Cu half‐cell with 0.4 g L^−1^ Ormosil + 1 M ZnSO_4_ electrolyte was 95.7%, considerably higher than that obtained with unmodified electrolyte (85.3%) (Figure 3f). Even the average CE of the half‐cells containing 0.1, 0.2 and 0.5 g L^−1^ Ormosil additive exceeded that of the blank cell (Figure S18). Thus, the introduction of Ormosil reduces side reactions of the zinc anode and increases the reversibility of Zn^2+^ in the electrolyte. The initial nucleation potential measured in the Zn||Cu half‐cell with both electrolytes decreased from 44 mV in 1 M ZSO to 34.8 mV after adding the electrolyte additive (Figure S19). Reversible zinc plating/stripping measurements using the Zn||Cu cell revealed that the Zn||Cu cell containing the 0.4 g L^−1^ Ormosil electrolyte exhibited stable, high plating/stripping efficiency at 5 mA cm^−2^ and 1 mAh cm^−2^, with an average CE of 99.74% (Figure 3g). In contrast, the blank Zn||Cu cell exhibited a lower CE with notable fluctuations over the subsequent 80 cycles, likely attributable to rampant dendrite growth and side reactions induced by H_2_O. The performance improvement after Ormosil addition demonstrates its stabilising effect on the zinc anode. The enhanced cycle life, higher rate capability and high CE, coupled with analysis of byproduct formation, support the crucial role of the Ormosil SEI layer in suppressing side reactions and promoting uniform zinc deposition, thereby facilitates an ultra‐stable zinc anode. The SEM image of the copper foil surface after 50 cycles at 2 mA cm^−2^ and 1 mAh cm^−2^, under conditions using Ormosil electrolyte and 1 M ZSO electrolyte (Figure 3h). For the half‐cell assembled using 1 M ZSO, a large number of plate‐like dendrites formed on the copper foil surface. With the electrolyte containing Ormosil, the Zn dendrites formed on the Cu foil were smaller, and Zn was uniformly deposited across the copper foil. This indicates that Ormosil can suppresses zinc anode protrusion formation.

### 2.3 Analysis of Mechanism

Combined electrochemical characterisation, including zeta potential, electrical double‐layer capacitance (EDLC) measurements and x‐ray photoelectron spectroscopy (XPS), revealed the mechanism of action of Ormosil.

**FIGURE 4:**
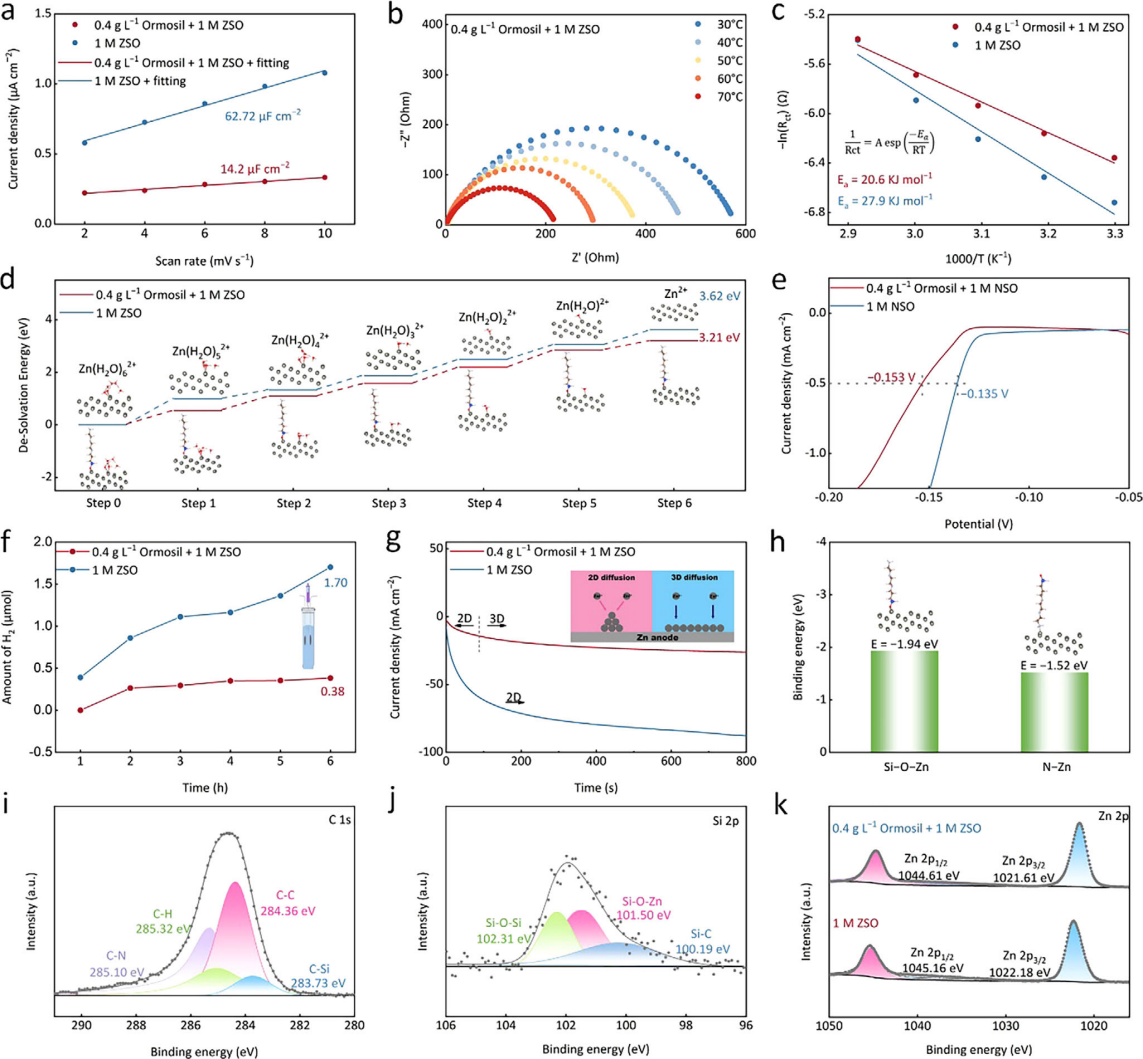
(a) EDL capacitance Zn anodes with/without Ormosil electrolyte. (b) Nyquist plots of the symmetrical cells with Ormosil addition at different temperatures. (c) De‐solvation activation energy tests. (d) DFT‐calculated desolvation energy barrier of [Zn(H_2_O)_6_]^2+^. (e) LSV curves of Zn anodes in sodium sulphate with/without Ormosil electrolyte. (f) Hydrogen evolution curve. (g) CA curve. (h) DFT calculations of the adsorption energies for Si−O−Zn and N−Zn. High‐resolution XPS spectra of (i) C 1s, (j) Si 2p and (k) Zn 2p.

Further investigation of the adsorption behaviour was carried out by obtaining differential capacitance data for Zn||Zn symmetric cells (Figure 4a and Figure S20). The electrical double‐layer capacitance of the Zn symmetric cell containing 0.4 g L^−1^ Ormosil electrolyte (14.2 µF cm^−2^) was lower than that with unmodified electrolyte (62.72 µF cm^−2^). Due to the high molecular rigidity and affinity for the Zn surface of the siloxane backbone in the Ormosil molecules, they anchor perpendicularly to the electrode surface. This increases the thickness of the Helmholtz layer, indicating a reduction in the charge consumed by the non‐Faradaic process, which decrease capacitance, thereby promote uniform zinc deposition [31]. The kinetics of Zn^2+^ deposition were evaluated by assessing the desolvation activation energy (E_a_) of Zn. The activation energy measurements were obtained by testing the impedance changes of the Zn||Zn cell at different temperatures (30°C–70°C). As shown in Figure 4b, Figure S21 and Table S2, the reaction kinetics accelerate with increasing temperature, leading to a gradual decrease in impedance. The desolvation activation energy (20.6 kJ mol^−1^) for the battery using Ormosil additives is significantly lower than that of the battery with an electrolyte of 1 M ZSO (27.9 kJ mol^−1^) (Figure 4c). The solvation structure of Zn^2+^ significantly influences its interfacial dynamics and cycle life. Further analysis of the regulation of Ormosil on Zn^2+^ solvation structure, the de‐solvation energies of typical [Zn(H_2_O)_6_]^2+^ complexes were calculated (Figure 4d). The density functional theory (DFT) calculated desolvation energy barrier of [Zn(H_2_O)_6_]^2+^ in the Ormosil‐additive electrolyte (3.21 eV) is significantly lower than that in the 1 M ZSO electrolyte (3.62 eV). The results suggest that the introduction of Ormosil effectively weakens the Zn^2+^−H_2_O bond strength, reduces the de‐solvation energy barrier, and thereby accelerates the de‐solvation process of Zn^2+^.

Linear sweep voltammetry (LSV) was used to assess the electrochemical stability window (Figure S22). The electrode in the 0.4 g L^−1^ Ormosil + 1 M ZnSO_4_ electrolyte exhibited a substantially higher HER potential than that in the blank electrolyte, indicating increased resistance to the HER in the 0.4 g L^−1^ Ormosil + 1 M ZnSO_4_ Ormosil electrolyte. Furthermore, 1 M Na_2_SO_4_ was substituted for the 1 M ZnSO_4_ electrolyte to prevent zinc deposition during measurements of the HER potential. As shown in Figure 4e, the addition of Ormosil resulted in a negative shift in the HER potential (−0.135 V for 1 M Na_2_SO_4_ vs. −0.153 V for the Ormosil‐containing electrolyte at −0.5 mA cm^−2^, indicating that the Ormosil additive considerably suppresses the electrochemical reduction of H_2_O molecules, leading to a slow HER. This shows that the Ormosil additive effectively broadens the electrochemical stability window, thereby suppressing the decomposition of H_2_O molecules during zinc stripping/plating. Comparison of the hydrogen evolution during zinc plating in the blank and Ormosil‐containing electrolytes demonstrated that the total hydrogen evolution in the symmetric cell with the blank electrolyte after 6 h of cycling was 1.70 µmol, compared to only 0.38 µmol in the Ormosil‐containing electrolyte (Figure 4f) [32]. This difference indicates a notable inhibitory effect of the Ormosil‐containing electrolyte compared to the blank electrolyte. The corrosion resistance was investigated using electrochemical impedance spectroscopy at open‐circuit voltage (Figure S23). The addition of Ormosil reduced the charge transfer impedance of the electrolyte. Further evidence confirms that the Ormosil additive effectively suppresses zinc dendrite growth. The Tafel curves revealed a suppressed corrosion current and elevated corrosion resistance potential for the zinc electrode in the Ormosil electrolyte (*E*_corr_ = −0.010 V, *I*_corr_ = 1.2739 mA cm^−2^ vs. *E*_corr_ = −0.013 V, *I*_corr_ = 2.2943 mA cm^−2^ for 1 M ZSO), which effectively suppressed corrosion and hydrogen evolution (Figure S24) [33].

Cyclic voltammetry analysis of zinc plating/stripping on the copper electrodes in both the blank and Ormosil‐containing electrolytes was used to elucidate the initial nucleation process. Compared with the blank electrolyte, the nucleation overpotential substantially reduced in the additive electrolyte (Figure S25). The critical nucleation radius (r) is inversely proportional to the absolute value of the overpotential (|η|). Thus, a larger nucleation overpotential leads to smaller, finely grained zinc nuclei, this promotes uniform zinc deposition on the zinc electrode facilitating the formation of a dense zinc layer [34]. Chronoamperometric (CA) tests were performed using a Zn||Zn cell at 150 mV (Figure 4g), the current in 1 M ZSO electrolyte decreased continuously in the initial stage, corresponding to a rapid increase in the electrode area during initial Zn nucleation. This is attributed to the rampant two‐dimensional (2D) diffusion of Zn^2+^, thereby leading to an uneven and loose deposition morphology. Conversely, for the modified electrolyte, the current indicated a transition to 3D diffusion after a brief period of 2D diffusion, favouring a uniform and dense deposition on the zinc electrode [35, 36]. Zeta potential measurements for the Zn anodes immersed in the blank electrolyte and Ormosil systems (Figure S26) show that Ormosil reduced the zeta potential from −1.38 to −2.81 mV, confirming its effective adsorption on Zn anode surface [37]. Furthermore, the calculated energy for the adsorption of Ormosil on Zn^2+^ was −1.76 eV. In contrast, the value for the Zn^2+^−H_2_O system is −1.32 eV (Figure S27), suggesting that the bond between Zn^2+^ and Ormosil is stronger.

The bonding behavior between the silane layer and the zinc foil was investigated using density functional theory calculations. The Si−O−Zn bond energy (1.94 eV) was significantly higher than the N−Zn bond (1.52 eV), confirming the formation of Si−O−Zn bonds (Figure 4h). The chemical composition and state of the Ormosil SEI layer were examined via XPS, with additional Ar^+^ sputtering at 10, 50, 100 and 300 s. Full‐spectrum measurements revealed Zn 2p, O 1s, N 1s, C 1s, S 2p and Si 2p peaks (Figure S28), Indicating the formation of an Ormosil SEI layer on the surface of the zinc foil. High‐resolution C 1s spectra of circulating Zn foil surface show distinct characteristic peaks at 285.10, 285.32, 284.36 and 283.73 eV, attributed to C−N, C−H, C−C and C−Si, respectively (Figure 4i). Peaks of Si−C (100.19 eV) and Si−O−Zn (101.50 eV) were observed in the Si 2p spectrum (Figure 4j). The presence of the Si−O−Zn bond confirms the chemical bonding between Zn and the Ormosil silane layer. In the high‐resolution C 1s XPS profile, the peak gradually diminished with increasing sputtering time and nearly vanished after 300 s, suggesting that the thickness of the Ormosil layer is several hundred nanometres (Figure S29). Furthermore, XPS analysis of the zinc anode after 50 cycles with blank electrolyte revealed distinct peaks of Zn, O and S on the anode surface (Figure S30). As revealed by the high‐resolution Zn 2p spectrum, the binding energies of Zn 2p_3/2_ and Zn 2p_1/2_ increased by 0.02 eV after Ormosil addition (Figure 4k), caused by chemical bonds forming between Zn and the Ormosil silane layer, specifically Si−O−Zn bonds [38, 39]. Additionally, peaks corresponding to Zn−O, −OH and S−O bonds were observed in the O 1s spectrum (Figure S31), whereas SO_4_^2−^ peaks were detected in the S 2p spectrum (Figure S32), attributable to byproducts. XPS analysis was performed following long‐term cycling, and the results confirmed the stable presence of Si−O−Zn bonds. The Ormosil siloxane network on the surface and the interfacial bonding maintained good structural integrity throughout the long‐term cycling (Figure S33). ATR‐FTIR and XPS analyses confirmed the formation of Si−O−Cu bonds on the Cu surface upon adsorption of Ormosil (Figure S34). This adsorbed layer modified the Cu surface and promoted uniform Zn nucleation, this synergistic optimization of the Zn−Cu dual interface accounts for the improved coulombic efficiency observed in the Zn||Cu battery [40].

### 2.4 In Situ Optical and SEM Observations

**FIGURE 5:**
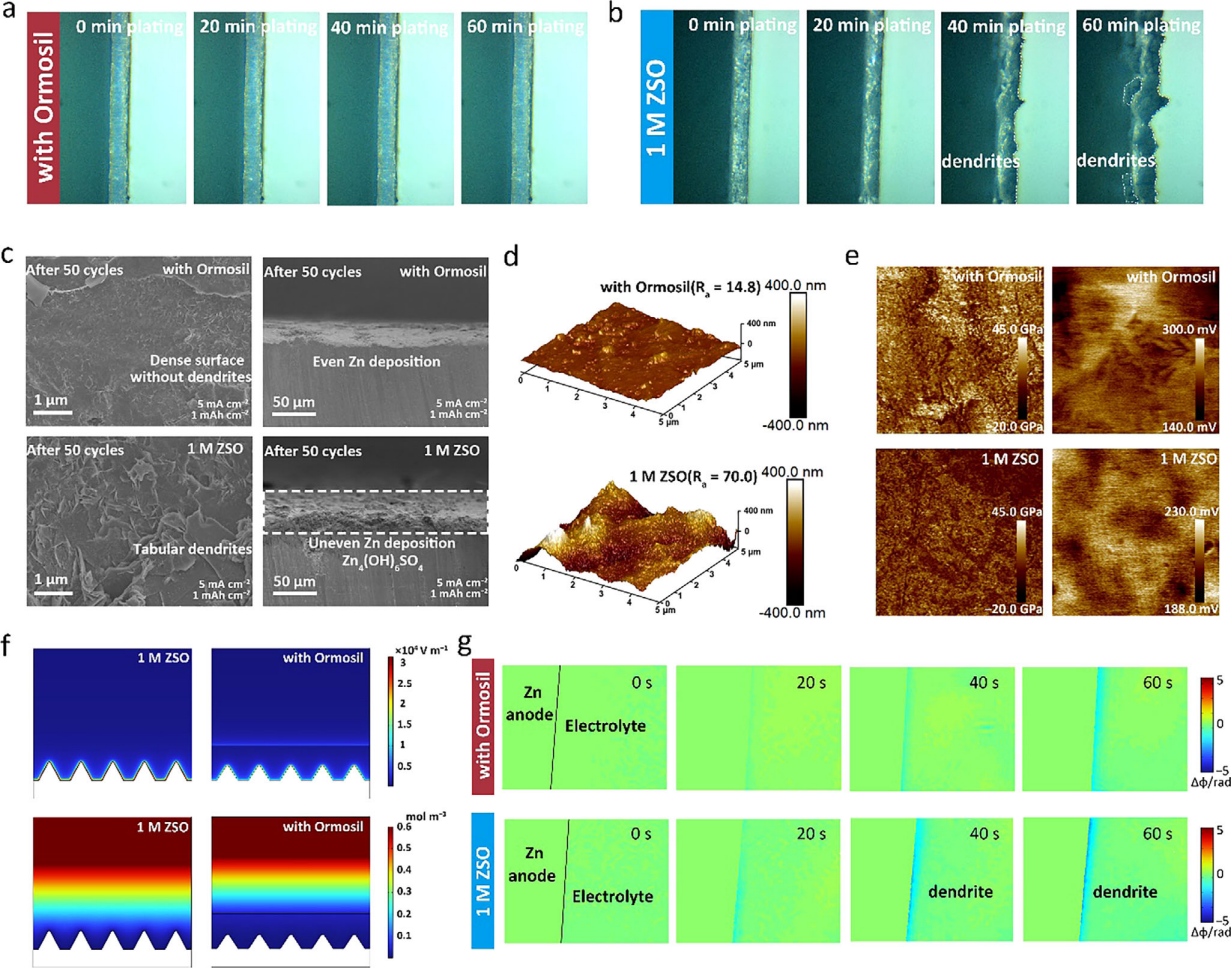
In situ optical microscopy images of Zn anode of zinc deposition in electrolyte (a) containing Ormosil and (b) in 1 M ZSO. (c) SEM image, (d) AFM image, (e) Young's modulus and surface potential distribution diagram images of Zn anode after 50 cycles. (f) Electric and concentration field distribution of Zn anode in electrolyte containing Ormosil and in 1 M ZSO. (g) Quantitative concentration phase maps of the deposition process at 1 mA cm^−2^.

The regulation of the interfacial microenvironment is most directly reflected in interfacial evolution. The evolution of zinc deposition was evaluated by observing the electroplating process using in situ optical microscopy at 1 mA cm^−2^. Without the Ormosil additive, dendrite growth was initiated after 20 min of deposition, resulting in uneven deposition and dendrite formation throughout the plating process. In the Ormosil system, Zn was uniformly deposited. No dendrites or bubbles were detected during the 60 min plating period (Figure 5a,b). The Zn electrode maintained a uniformly flat surface throughout the deposition process, demonstrating the high efficacy of Ormosil in suppressing the occurrence of side reactions and promoting uniform Zn deposition. The SEM images of the deposits on the zinc foil after cycling at different current densities show loose and uneven zinc deposition with distinct dendrite formation at all current densities in the blank electrolyte. In contrast, in the Ormosil‐containing electrolyte, the zinc deposits were dense and accumulated in a compact manner (Figure S35). After 50 cycles of Zn||Zn cell, the SEM images of the Zn electrode were acquired (Figure 5c). The cycled Zn anode surface from the blank electrolyte had many flakes and distinct protrusions, attributed to uneven Zn^2+^ deposition and interfacial side reactions. In contrast, the addition of Ormosil resulted in a dense, flat surface of the zinc foil without byproducts. An atomic force microscope (AFM) was used to study the surface morphology of zinc electrode. The surface of the electrode in the Zn||Zn cell cycled in 1 M ZSO electrolyte became rough and uneven, with a maximum height of 417 nm (R_a_ = 70.0) (Figure 5d), whereas in the electrolyte containing Ormosil, the maximum height was only 130 nm (R_a_ = 14.8). As a key indicator for evaluating SEI strength, the Young's modulus and potential distribution map on the Zn anode surface were investigated after 50 cycles. The average Young's modulus of the Zn anode in the 0.4 g L^−1^ Ormosil + 1 M ZSO electrolyte after 50 cycles was significantly higher than that in the 1 M ZSO electrolyte, indicating superior mechanical properties. This demonstrates that the uniform distribution of the Ormosil silane layer on the Zn anode surface suppresses dendrite growth and produces a more uniform zinc plating layer. The surface potential distribution diagram of the Zn electrode cycled 50 cycles in 0.4 g L^−1^ Ormosil + 1 M ZSO exhibits a more uniform interfacial electric field (potential difference < 47.1 mV). For the Zn anode cycled 50 cycles in 1 M ZSO, a highly non‐uniform surface morphology with an uneven space charge field (exhibiting a higher potential difference of 107.6 mV) (Figure 5e) [41, 42].

To further investigate the effects of Ormosil on deposition and stripping, Zn^2+^ concentration and electric field distribution at the electrode interface were investigated by COMSOL finite element simulations (Figure 5f) [43]. A distinct protrusion was observed on the zinc anode surface in 1 M ZSO, with the electric field concentrated at the surface tip, resulting in an uneven local electric field intensity. Additionally, from the top region to the bottom region of the Zn anode, Zn^2+^ concentration rapidly declines, leading to a significant concentration gradient and fluctuations near the surface. This leads to preferential Zn^2+^ deposition and dendritic growth, ultimately causing battery short circuits. Conversely, the presence of Ormosil additives significantly reduces the electric field strength of Zn anode surface. This leads to smaller zinc dendrites forming on the electrode surface. Simultaneously, a lower Zn^2+^ concentration gradient is achieved at the electrode surface, Due to the protonation and deprotonation of −NH_2_, the local pH decreases during the early stages of dendrite nucleation, leading to an accumulation of −NH_3_^+^, which electrostatically repels Zn^2+^ and thereby inhibits subsequent zinc dendrite growth. In addition, in situ electrochemical digital holography was used to measure changes in interfacial ion concentration during the deposition and exfoliation processes. Blue indicates a decrease in interfacial ion concentration, while red indicates an increase. Within 60 s of deposition, significant localized depletion of Zn^2+^ was observed in the blank electrolyte, resulting in an uneven ion concentration gradient and, consequently, non‐uniform deposition. In the electrolyte containing Ormosil, uniform interfacial ion distribution was observed, indicating uniform Zn deposition (Figure 5g). During the initial stage of stripping, significant stripping occurred in the electrolyte containing the Ormosil additive. However, as stripping progressed, the growth of ion concentration on the zinc anode surface was suppressed due to electrostatic repulsion from −NH_3_^+^. In contrast, for the blank electrolyte, Zn^2+^ concentration became non‐uniform at the interface and in the vicinity of the electrolyte starting at 60 s (Figure S36). After prolonged cycling, the surface of the separator treated with an electrolyte containing Ormosil additives remained intact, with no significant dendrite growth, whereas the separator treated with 1 M ZSO electrolyte exhibited significant byproduct deposition and zinc dendrites (Figure S37).

### 2.5 Electrochemical Performance of Full Cells

**FIGURE 6:**
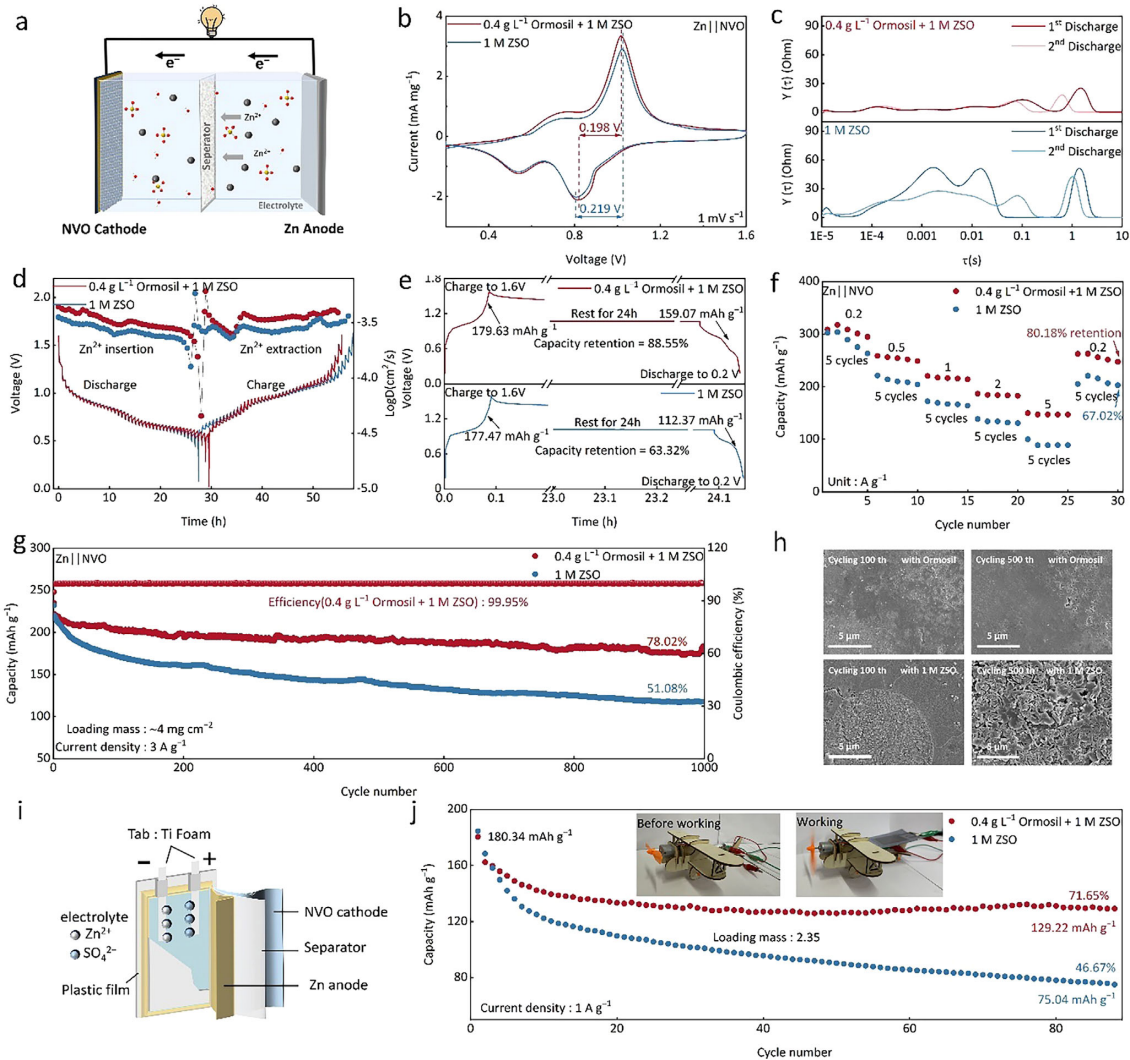
(a) Zn||NVO full cell schematic illustration. (b) CV curve. (c) The DRT curves of Zn||NVO cells. (d) GITT curves and calculated diffusion coefficients. (e) Self‐discharge tests. (f) Rate performance. (g) Cycling performance of the Zn||NVO cell at 3 A g^−1^. (h) SEM images of both electrodes in full cells after 100 cycles and 500 cycles. (i) Schematic illustration of pouch cell. (j) Cycling performance of pouch cells.

To validate the practical applicability of the strategy of adding Ormosil, A NaV_3_O_8_ (NVO) cathode was synthesized based on the literature. Figure S38 shows the SEM image of the NVO material, which exhibits a flocculent morphology. Figure S39 shows the XRD pattern of the synthesised NVO. Photographs of the NVO‐coated electrode after immersion in the blank and Ormosil‐containing electrolytes for 1, 5 and 10 days reveal that in the blank electrolyte, the electrode dissolved and detached, whereas the electrode immersed in the Ormosil‐containing electrolyte showed no considerable changes (Figure S40) [44]. Figure 6a shows a schematic structure of the Zn||NVO cell. The CV curves of the Zn||NVO cells with the blank and Ormosil‐containing electrolytes (Figure 6b) demonstrate that the use of Ormosil reduces the voltage polarisation and increases the current response, indicating enhanced reaction kinetics and improved reversibility, plausibly attributed to increased chemical stability. The two pairs of reversible redox peaks at 0.85/1.06 V and 0.55/0.77 V can be attributed to changes in the valence state of vanadium ions, specifically the transition from V^4+^ to V^3+^ and from V^5+^ to V^4+^, respectively [45]. After introducing the Ormosil additive, the SEI layer formed by Zn^2+^ and the Ormosil additive reduced the charge transfer resistance, as also confirmed by electrochemical impedance spectroscopy (EIS) measurements of the Zn||NVO cell (Figure S41). Additionally, distribution of relaxation times (DRT) analysis was performed during the first and second discharge cycles (Figure 6c). The DRT data revealed four relaxation times (τ_r_): adsorption of Zn^2+^ (*R_ads_*) (10^−2^), surface migration (*R_mig_*) (10^−1^), charge transfer (*R_ct_*) (10^0^), and diffusion (*R_diff_*) (10^1^) [46]. The full cell using 1 M ZSO electrolyte exhibited higher Y(τ) values with a significantly increased charge transfer impedance value, indicating byproduct generation. However, the Y(τ) curve of the full cell incorporating the Ormosil additive showed almost no change. This demonstrates that Ormosil dynamically adjusts the pH level, resulting in a more stable interfacial environment. The diffusion coefficient of Zn^2+^ was measured using galvanostatic intermittent titration technique (GITT) [47]. As shown in Figure 6d, consistent with EIS results, the full cell incorporating the Ormosil electrolyte additive exhibited a higher Zn^2+^ diffusion coefficient. This indicates that Ormosil promotes rapid Zn^2+^ transfer and reduces side reactions. Secondly, the self‐discharge behavior of the full cell was investigated to evaluate internal stability. As shown in Figure 6e, the cell incorporating the additive Ormosil exhibited a high capacity retention of 88.55%. In contrast, the cell using 1 M ZSO demonstrated a retention of 63.32% after 24 h of storage. Corrosion of the zinc negative electrode causes a local increase in pH, which promotes the dissolution of the positive electrode material; the dissolved metal ions migrate to the surface of the Zn, further accelerating zinc corrosion. Therefore, the attenuation of self‐discharge is the result of the interaction between the self‐corrosion of the negative electrode and the dissolution of the positive electrode [48]. This indicates that the formed SEI film layer protects the Zn anode from potential side reactions induced by active materials in the electrolyte.

Figure 6f demonstrates that the Zn||NVO cell with the Ormosil‐modified electrolyte has superior rate performance. Compared to the cell containing 1 M ZSO, consistently higher capacities were observed at each tested current density. Upon returning to 0.2 A g^−1^, a capacity of 260 mAh g^−1^ was maintained, as clearly evident from the capacity‒voltage curves (Figure S42). Zn||NVO cells employing the Ormosil electrolyte showed an increased specific capacity. At 1 A g^−1^, the specific capacity remained over 189.6 mAh g^−1^ after 200 cycles (capacity retention = 70.35%), substantially higher than that of the cell with 1 M ZSO (129.06 mAh g^−1^; capacity retention = 48.84%) (Figure S43). Furthermore, as shown in Figure 6g, at 3 A g^−1^, the specific capacity of the battery with Ormosil electrolyte remained above 180.09 mAh g^−1^ after 1000 cycles, with a capacity retention rate of 76.60%. The full cell with the blank electrolyte resulted in rapid capacity decay during cycling, with a capacity of only 118.52 mAh g^−1^ after 1000 cycles and achieving a capacity retention rate of 51.08%. As shown in Figure 6h, SEM images of zinc anodes from full cells with and without Ormosil additive after 100 and 500 cycles reveal porous Zn dendrites and byproducts on the Zn surface of the 1 M ZSO‐containing cell. In contrast, the zinc anode surface of the cell using Ormosil additive remains flat and dense even after 500 cycles, consistent with its excellent cycling stability. The XRD patterns and SEM images of the NVO cathode sheets after full cell cycling show that the cathode sheets in the full cells using Ormosil maintained a good morphology, whereas those in the full cells using 1 M ZSO electrolyte exhibited significant crystal structure degradation and the formation of noticeable byproducts, demonstrating that the addition of Ormosil significantly improves the structural stability of the cathode sheets (Figures S44 and S45). Furthermore, at 5 A g^−1^, cyclic testing was conducted using a 0.4 g L^−1^ Ormosil + 1 M Zn(OTf)_2_ electrolyte (Figure S46). After 1000 cycles, the Zn||NVO cell incorporating the Ormosil additive still with a high specific capacity (163.94 mAh g^−1^). This demonstrates the suitability of this additive. A Zn||MnO_2_ cell was assembled to verify the universality of Ormosil. The full cell incorporating the Ormosil additive exhibited superior cycling stability; at 0.1 A g^−1^, after 100 cycles, the full cell using the Ormosil maintained a high specific discharge capacity of 137.83 mAh g^−1^, whereas the full cell using 1 M ZSO exhibited a faster capacity decay (47.84 mAh g^−1^) (Figure S47).

To further assess the practical use of Ormosil additives, we assembled pouch cells and conducted electrochemical characterization. NVO cathode material was cut into 4 × 5 cm rectangles and assembled into pouch cells, with a simulated image of the pouch cell shown in Figure 6i. The pouch cell has an open‐circuit voltage of 1.338 V (Figure S48). At 1 A g^−1^, the pouch cell using Ormosil maintained a capacity of 129.22 mAh g^−1^ after 90 cycles, and capacity retention rate of 71.65%. This significantly outperformed the pouch cell using 1 M ZSO (specific capacity: 75.04 mAh g^−1^, capacity retention rate: 46.67%) (Figure 6j). Additionally, the pouch cell successfully powered the successfully powered the propeller of the model biplane. Ormosil plays a strengthening role in the zinc anode, enhancing the cycling performance and specific capacity.

## 3 Conclusions

Herein, the electrolyte additive Ormosil was employed to form SEI layer on zinc anodes, achieving outstanding electrochemical performance in AZIBs. Combined experimental characterization and theoretical calculations indicate that –NH_2_ modulates the interfacial microenvironment through protonation/deprotonation. ─NH_2_ groups can modulate the pH at the electrode‐electrolyte interface, provide appropriate alkalinity, and facilitate strong interactions with Zn^2+^. Inhibit Zn^2+^ aggregation during the early stages of dendrite growth; The formation of Si─O─Zn bonds enable the formation of durable bonds between zinc and the Ormosil layer, while the Si─O─Si three‐dimensional network forms a physical barrier that regulates ion flux and blocks water clusters. Together, these two mechanisms synergistically achieve multidimensional kinetic and thermodynamic optimization of zinc deposition behavior, not only suppress chemical corrosion but also promote uniform plating/stripping. The Ormosil system exhibits outstanding electrochemical performance, enabling cycling for approximately 1000 h at 10 mA cm^−2^ and 2 mAh cm^−2^ in a symmetric cell, and CE of 99.74% over 500 cycles at 5 mA cm^−2^ and 1 mAh cm^−2^ in a half‐cell. With Ormosil additive, the Zn||NVO full cell exhibits enhanced electrochemical performance. After 1000 cycles at 3 A g^−1^, the modified cell still demonstrates a specific capacity of approximately 180.09 mAh g^−1^. Ormosil addition represents an effective new approach to achieving high‐performance.

## Author Contributions

**Chao Lai**: data curation. **Haiyang Wu**: data curation. **Lina Pan**: writing – original draft. **Peng Huang**: data curation, writing – review and editing. **Boyu Yuan**: data curation. **Yaqian Lan**: conceptualization, writing – review and editing.

## Conflicts of Interest

The authors declare no conflicts of interest

## Supporting information




**Supporting File**: advs76526‐sup‐0001‐SuppMat.docx.

## Data Availability

The data that support the findings of this study are available from the corresponding author upon reasonable request.
