# Supplementary material for: A Bifunctional Organosilane Additive for Dynamic pH Regulation and Interfacial Protection in Aqueous Zinc‐Ion Batteries
Source: Adv Sci (Weinh). 2026 Jul 11;13(55):e76526. doi: 10.1002/advs.76526 (PMC13355892; doi:10.1002/advs.76526)
Supplement: Supplementary file 1 [file ADVS-13-e76526-s001.docx]

Supporting Information

**A Bifunctional Organosilane Additive for Dynamic pH Regulation and Interfacial Protection in Aqueous Zinc-Ion Batteries**

*Lina Pan^a†^, Haiyang Wu^a†^, Peng Huang^a^*, Boyu Yuan^c^, Chao Lai^a^, and Yaqian Lan^b^**

^a^School of Chemistry and Materials Science, Jiangsu Normal University, Xuzhou, 221116, P. R. China

^b^Guangdong Provincial Key Laboratory of Carbon Dioxide Resource Utilization, South China Normal University School of Chemistry, Guangzhou, 510006, P. R. China

^c^School of Physics and Electronic Engineering, Jiangsu Normal University, Xuzhou, 221116, P. R. China

Corresponding authors. E-mail: huangpeng@jsnu.edu.cn; yqlan@m.scnu.edu.cn

^†^These authors contributed equally to this work and should be considered co-first authors.

**Materials:**

The purity Zn foil is 99.99%, The diameter of the Zn plate is 12 and 10 mm and the thickness is 100 μm. The purity Cu foil is 99.99% and the thickness is 12 μm. ZnSO_4_·7H_2_O, V_2_O_5_, Zn(CF_3_SO_3_)_2_ were purchased from Sinopharm Chemical Reagent Co., Ltd. NaCl, N-methylpyrrolidone (NMP), ethanol (99.95%), ATS 3-[2-(2-aminoethylamino)ethylamino]propyl-trimethoxysilane), dimethylformamide (DMF), were purchased from Shanghai Aladdin Biochemical Technology Co., Ltd. Super P were purchased from Henan Jinghong (Group) Co., Ltd.

**Preparation of Electrolytes:**

Stir 4.397 g of TEOS and 5.603 g of ATS in 10 mL of deionized water for 30 minutes. Obtain the TEOS/ATS/water mixture solution and add HCl to adjust the pH to 4. The solution obtained by extracting and removing unreacted ATS is the Ormosil solution. Dissolve 28.756 g of zinc sulfate heptahydrate and transfer the solution to a 100 mL volumetric flask. Make up to volume with deionized water to prepare a 1 mol L^−1^ zinc sulfate solution (blank electrolyte). For the Ormosil-based electrolyte, 0.001 g, 0.002 g, 0.004 g, and 0.005 g of Ormosil were added to 10 mL of solution to obtain electrolytes containing 0.1 g L^−1^, 0.2 g L^−1^, 0.4 g L^−1^, and 0.5 g L^−1^ of Ormosil, respectively.

**Electrochemical measurement:**

Aqueous zinc ion batteries are housed within coin cells (CR2032). The zinc plate has a diameter of 12 mm and a thickness of 0.1 mm. The separator is a glass fiber membrane (Whatman GF/D−90 nm). A zinc plate with a diameter of 12 mm serves as the counter electrode. The half-cell employs a copper plate with a diameter of 10 mm as the negative electrode. The full cell utilizes NaV_3_O_8_ prepared by dissolving 1.0 g of V_2_O_5_ into 15 mL of 2 mol sodium chloride aqueous solution. After stirring for 72 hours, the resulting brown suspension was washed with distilled water, centrifuged, and dried overnight in a vacuum drying oven at 80 °C. The NVO anode was prepared by pressing a mixture of NVO, Super P, and polytetrafluoroethylene (PTFE) binder in a mass ratio of 7:2:1. Zn||Zn symmetric cells, Zn||Cu half-cells, and Zn||NaV_3_O_8_ (Zn||NVO) full cells underwent electrochemical performance testing on the Newell battery testing system. Tafel, linear sweep voltammetry (LSV), cyclic voltammetry (CV), and electrochemical impedance spectroscopy (EIS) tests were conducted on the CHI760E electrochemical workstation (Shanghai Huachen Instruments Co., Ltd.). A three-electrode system was employed to test the three electrodes: Ag/AgCl as the reference electrode, platinum electrode as the counter electrode, and zinc foil as the working electrode. The CA curve was measured at a constant potential (150 mV). The frequency range for electrochemical impedance spectroscopy was 0.01–100000 Hz.

**Characterization:**

The microstructure of zinc foil was examined using a Scanning Electron Microscope (SEM) (SU8010, Hitachi, Japan), AFM (Bruker Dimension Icon), KPFM (Bruker), Young’s (Bruker). Morphological changes during zinc foil deposition were characterized using in-situ optical microscopy images obtained with XSP-20 microscope. The crystalline structure of the Zn anodes was characterized by X−ray diffraction (XRD) on Bruker (Germany, D8 ADVANCE). Energy Dispersive Spectrometry (EDS) instrument (EDAX, PW9900) was used to analyze the types and distribution of elements on the electrode surface. The composition of the Zn anodes was characterized by XPS (K−Alpha of Thermo Fisher Company, USA, the zinc electrode was etched by Ar^+^ sputtering at the energy density of 1000eV and the current of mid), ATR-FTIR (Thermo Fisher Sientific, IS50), Raman (Horiba he800). The contact angle tester (KRUSS DSA25) was used to test the wettability of electrolyte on the surface of Zn electrode.

**Ionic conductivity calculation:**

The ionic conductivity(σ) of various electrolytes that was tested in the titanium foil symmetric cells, are calculated as follows:

$\sigma= \frac{L}{\mathrm{SR}}$	(1)

where L is the distance between two parallel stainless-steel plates, S is the area of the stainless-steel plate and R is the ohmic impedance.

**Electric double-layer capacitance (EDLC) calculation:**

The capacitance (C) was determined by the linear relationship between capacitive current (i_c_) and scan rate (v), which can be obtained from the slope of the i_c_ versus v graphs. Therefore, the EDL capacitance was calculated through the following equation:

$C =\frac{i_{c}}{v}$	(2)

Where i_c_ refers to the capacitive currents in CV scans. Here, we chose：$i_{c}=\frac{i_{0v}+\left( -i_{0v-} \right)}{2}$, meaning half value of current difference during forward scan and negative scan at 0 V. v refers to the scan rates of CV tests.

**Measurement of Activation Energy (E_a_):**

EIS experiment was carried out with a frequency range from 10^5^ to 10^−2^ Hz with the perturbation of 5 mV. According to the time-dependent EIS measurements, the desolvation process of Zn^2+^ could be evaluated by the activation energy in the Arrhenius equation:

$\frac{1}{R_{ct}}=A esp\left( \frac{-E_{a}}{\mathrm{RT}} \right)$	(3)

In this case, *R_ct_* represents the interfacial resistance, A is the frequency factor, R is the gas constant, and the absolute temperature is denoted by T.

**Computational details:**

All calculations were performed using the plane-wave periodic DFT method in the Vienne ab initio simulation package (VASP).^[1-2]^ The generalized gradient approximation (GGA) of Perdew–Burke−Ernzerh (PBE) exchange-correlation functional was used.^[3]^ The projector augmented wave (PAW) method is used to describe the electron-ion interaction.^[4-5]^ The vdW-D3 method developed by Grimme was employed to describe the van der Waals interaction.The cutoff energy was set as 400 eV to ensure the accurate energies. The Gaussian electron smearing method is used by σ = 0.05 eV. The DFT lattice parameters for the Zn(101) bulk are a = 16.857 Å, b = 13.325 Å, c = 24.532 Å,^[6]^ The surface of (101) was modeled by periodic slabs with *p*(2×2) unit cell. The vacuum region with a minimum thickness of 20 Å was included between the slabs. A 3×3×1 Monkhorst−Pack k-point grid was used for sampling the Brillouin zone. The bottom one atomic layer of the supercell was fixed and other layers were full relaxed to reduce the computational cost. The geometry optimization was done when the energy difference was lower than 10^−4^ eV and the convergence criterion on forces smaller than 0.05 eV/Å, and a maximum displacement of 1.0×10-2 Å were considered.

The electrochemical simulation of zinc negative was carried out by COMSOL Multiphysics. Tertiary Current Distribution (electroneutrality) physics interface was used to model the transport behavior of charged species near the zinc electrode. The governing equation is Nernst-Planck equation:

$\frac{\partial c_{i}}{\partial t}=\nabla\cdot(D_{i}\nabla c_{i}+\frac{{D_{i}z}_{i}}{RT}Fc_{i}\nabla\phi)$	(4)

Where $c_{i}$, $z_{i}$, and $D_{i}$ are the concentration, charge number and diffusion coefficient of species $i$ respectively, $\phi$ is electrolyte potential, $R$ and $F$ are ideal gas constant and Faraday constant respectively.

The concentration of species that are accounted for were primary reactive species Zn^2+^ and primary counterion SO_4_^2−^ The reduction reaction kinetic of Zn^2+^ were described by Butler−Volmer equation. In the Butler–Volmer expression, the anodic and cathodic transfer coefficients were both set to 0.5. The reference exchange current density was set to 100 A m^−2^. the initial and bulk concentrations of Zn^2+^ and SO_4_^2−^ were both set to 1000 mol m^−3^, corresponding to 1 M ZnSO_4_. The temperature was fixed at 298.15 K. The diffusion coefficients of Zn^2+^ and SO_4_^2−^ in the bulk electrolyte were set to 7.15 × 10 and 1.070 × 10^−9^ m^2^ s^−1^, respectively, according to reported ionic diffusion data1. The equilibrium potential of zinc plating reaction was defined using the Nernst equation, with a standard electrode potential of 0 V. The electrode was operated with an applied potential of −0.27 V below equilibrium potential.

The model was a 2-dimensional geometry with width 120 um and height 100 um. The bottom edge represents electrode surface and the top edge represents bulk solution. Five dendrite seeds were placed on the bottom edge in equilateral triangular shape with edge length of 15 um. In contrast with the bare electrode, the additive was represented by an array of non-permissible rectangle with 2 um width, 4 um height and 1 um distance in-between.

Physics controlled mesh was used to discretize the geometry. The maximum element size near the electrode interface was set to 0.2 um, while the maximum element size in the bulk electrolyte was set to 2 um. The time-dependent solver used a fully coupled nonlinear scheme with a relative tolerance of 10^−3^. The local Zn^2+^ concentration and ionic current density were extracted to evaluate the uniformity of Zn^2+^ transport and Zn deposition.

**Results and Discussion**

**Figure S1.** Photos of Ormosil.

**Figure S2.** Cycling tests for Zn||Zn symmetric cells in 0.05 and 1 g L^−1^ Ormosil electrolytes were conducted at 10 mA cm^−2^ and 2 mAh cm^−2^.

**Figure S3.** Comparison of pH of electrolytes with different Ormosil concentrations.

**Figure S4.** Contact angle tests of Zn foils in electrolytes of 1 M ZSO and 1 M ZSO with varied concentrations of Ormosil.

**Figure S5.** Solvent separated ion pair (SSIP, [Zn^2+^(H_2_O)_6_SO_4_^2−^]) and contact ion pair (CIP, [Zn^2+^(H_2_O)_5_OSO_3_^2−^]) in ZnSO_4_ electrolyte.

**Figure S6.** The ATR-FTIR spectrum of Zn after cycling with Ormosil.

**Figure S7.** SEM image of zinc foil after cycling and corresponding EDS profile.

**Figure S8.** The elemental mappings of the Cu foil surface 10 s of plating in the Zn||Cu half-cell added with Ormosil additive at the current density of 1 mA cm^−2^.

**Figure S9.** SEM images of Zn anode after immersion in different Ormosil concentrations electrolytes for 7 d.

**Figure S10.** XRD pattern of the Zn foils after immersion in electrolytes without/with Ormosil additive for 7 d.

**Figure S11.** ATR-FTIR spectra of before and after cycling of electrolyte containing 0.4 g L^–1^ Ormosil + 1 M ZSO.

**Figure S12.** ATR-FTIR spectra in the range of 2800−3800 cm^−1^ of 1 M ZSO、0.4 g L^−1^ Ormosil + 1 M ZSO and H_2_O.

**Figure S13.** Radial distribution function of the Zn−O(H_2_O) in 1 M ZSO electrolyte.

**Figure S14.** 3D snapshot of molecular dynamics image simulation cells in the 1 M ZSO electrolyte.

**Figure S15.** Lifespans of Zn||Zn symmetric cells using 1 M ZSO and Ormosil electrolytes were conducted at a current density of 5 mA cm^−2^ with a fixed capacity of 1 mAh cm^−2^.

**Figure S16.** Cycling tests for Zn||Zn cells at 1 mA cm^−2^ and 5 mAh cm^−2^.

**Figure S17.** Schematic illustration of the proposed “reservoir half-cell” for screening Zn plating/stripping CE.

**Figure S18** Voltage vs. time profile for Zn||Cu cells using electrolytes with 0.1, 0.2, 0.5 g L^−1^ Ormosil additive.

**Figure S19.** Initial Zn nucleation and growth overpotential of Zn||Cu cells in different electrolytes.

**Figure S20.** Cyclic voltammetry curves for Zn symmetric cells in the electrolytes a) without or b) with Ormosil at different scan rate.

**Figure S21.** Electrochemical impedance spectroscopy curves of the symmetric cells with 1 M ZSO at various temperatures.

**Figure S22.** The HER LSV curves tested in the electrolytes with/without Ormosil.

**Figure S23.** Nyquist plots of the symmetric cells with electrolytes with/without Ormosil.

**Figure S24.** Tafel polarization plots of Zn foils in different electrolytes.

**Figure S25.** CV curve of Zn||Cu half-cell.

**Figure S26.** Zeta potential of different electrolytes.

**Figure S27.** The adsorption on Zn^2+^ of different electrolytes.

**Figure S28.** High-resolution XPS spectra.

**Figure S29.** The high-resolution XPS spectrum of C 1s of the surface of the Zn anode after 50 cycles and the corresponding Ar^+^ sputtering measurements at different times.

**Figure S30.** High-resolution XPS spectra of Zn anode in the 1 M ZSO electrolyte after 50 cycles.

**Figure S31.** High-resolution XPS spectra of O 1s of the Zn anode in the 1 M ZSO electrolyte after 50 cycles.

**Figure S32.** High-resolution XPS spectra of S 2p of the Zn anode in the 1 M ZSO electrolyte after 50 cycles.

**Figure S33.** High-resolution XPS spectra of Si 2p of the Zn anode in the 0.4 g L^−1^ Ormosil + 1 M ZSO electrolyte after 500 cycles.

**Figure S34.** a) ATR-FTIR and b) Si 2p XPS spectra of Cu foil after cycling of Zn||Cu batteries using Ormosil additives.

**Figure S35.** SEM images of zinc anodes after deposition at different current densities.

**Figure S36.** Quantitative concentration phase maps of the stripping process at 1 mA cm^−2^.

**Figure S37.** Photomicroscopic and SEM images of the separator after cycling using a, b) 1 M ZSO and c, d) 0.4 g L^−1^ Ormosil + 1 M ZSO electrolytes.

**Figure S38.** The SEM of NVO powder.

**Figure S39.** XRD patterns of commercial NaV_3_O_8_·1.5H_2_O power.

**Figure S40.** Photos of NVO cathode immersed in 1 M ZSO and 0.4 g L^−1^ Ormosil + 1 M ZSO electrolytes for different days.

**Figure S41.** Nyquist plot of the Zn||NVO full cell.

**Figure S42.** Discharge/charge curves of Zn||NVO full cells from 0.2 A g^−1^ to 5 A g^−1^ using a) 0.4 g L^−1^ Ormosil + 1 M ZSO electrolyte and b) 1 M ZSO electrolyte.

**Figure S43.** Cycling performance of the Zn||NVO full cell at 1 A g^−1^.

**Figure S44.** SEM images of the NVO cathode after 500 cycles in Zn||NVO cell using a) 0.4 g L^–1^ Ormosil + 1 M ZSO electrolyte and b) 1 M ZSO electrolyte at 3 A g^–1^.

**Figure S45.** XRD of NVO after 500 cycles using 0.4 g L^−1^ Ormosil + 1 M ZSO electrolyte and 1 M ZSO electrolyte.

**Figure S46.** Cycle performance of Zn||NVO full cell using an electrolyte containing 0.4 g L^–1^ Ormosil + 1 M Zn(OTf)_2_ and 1 M Zn(OTf)_2_ at 5 A g^–1^.

**Figure S47.** Cycle performance of Zn||MnO_2_ full cell at 0.1 A g^–1^.

**Figure S48.** Open circuit voltage of Zn||NVO pouch full cell.

**Table**

**Table S1.** Literature survey of previous reports of surface-modified Zn anodes in aqueous zinc batteries.

| **Electrolyte** | **Current density(mAcm^−2^)** | **Capacity (mAh cm^−2^)** | **Life (h)** | **Ref** |
| --- | --- | --- | --- | --- |
| 1 M ZnSO_4_ + DTPA | 2 | 1 | 800 | ^[7]^ |
| 3 M Zn(OTf)_2_ + PNM | 10 | 1 | 640 | ^[8]^ |
|  | 5 | 1 | 1500 |  |
| ZnSO_4_ + Zinc p-Phenolsulfonate | 5 | 2.5 | 375 | ^[9]^ |
| 1 M ZnSO_4_ + D-Phenylalanine | 5 | 1 | 1200 | ^[10]^ |
| 2 M ZnSO_4_ + Na_3_NTA | 10 | 2.5 | 680 | ^[11]^ |
| 1 M ZnSO_4_ + Ce_2_(SO_4_)_3_ | 5 | 1 | 700 | ^[12]^ |
| ZnSO_4_ + xylo-oligosaccharide | 1 | 1 | 950 | ^[13]^ |
|  | 5 | 5 | 320 |  |
| 1 M ZnSO_4_ + Tripropylene glycol | 1 | 0.5 | 1100 | ^[14]^ |
| 2 M Zn(ClO_4_)_2_ + TS | 1 | 1 | 1000 | ^[15]^ |
| 1 M ZSA | 2 | 0.5 | 1400 | ^[16]^ |
| 2 M ZS + 0.1 M PL-As | 0.5 | 0.25 | 848 | ^[17]^ |
| 2 M ZnSO_4_ + [BMIM]PF_6_ | 1 | 1 | 800 | ^[18]^ |
|  | 4 | 0.5 | 1000 |  |
| 2 M ZnSO_4_ + DA | 10 | 1 | 700 | ^[19]^ |
|  | 2 | 1 | 1000 |  |
| 2 M ZnSO_4_ + SDA | 1 | 1 | 2300 | ^[20]^ |
| This work | 10 | 2 | 1000 |  |
|  | 5 | 1 | 1800 |  |
|  | 1 | 1 | 2500 |  |

**Table S2.** Charge transfer resistance of symmetrical cell with 1 M ZSO and 0.4 g L^−1^ Ormosil + 1 M ZSO at various temperatures.

| **Temperature** | **30 ℃** | **40 ℃** | **50 ℃** | **60 ℃** | **70 ℃** |
| --- | --- | --- | --- | --- | --- |
| 1 M ZSO | 828 Ω | 675 Ω | 496.2 Ω | 362.3 Ω | 222.6 Ω |
| 0.4 g L^−1^ Ormosil + 1 M ZSO | 577.3 Ω | 473.5 Ω | 378.2 Ω | 295.2 Ω | 220.7 Ω |

**Table S3.** Cost of raw materials for electrolyte preparation.

| Reagent | Price | Power consumption per button cell | Cost per button cell | Total cost per button cell（Ormosil） | Total cost per button cell  （1 M ZSO） |
| --- | --- | --- | --- | --- | --- |
| Tetraethyl orthosilicate | 33 USD L^−1^ | 18 μL | 5.94×10^−4^ USD | 0.0120 USD | 0.00048 USD |
| 3-[2-(2-aminoethylamino) ethylamino]propyl-trimethoxysilane) | 520 USD L^−1^ | 21 μL | 0.0109 USD |  |  |
| HCl | 80 USD L^−1^ | ~0 μL | ~0 USD |  |  |
| ZnSO_4_·7H_2_O | 14 USD kg^−1^ | 3.4507 ×10^−5^ kg | 0.000483 USD |  |  |

Data source: Prices on the Aladdin online platform. URL: https://www.aladdin-e.com/ Time：June 6, 2026.

**References**

[1]	G. Kresse, J. Furthmüller, *Comput. Mater. Sci.* **1996**, *6* (1), 15.

[2]	*Phys. Rev. B* **1996**, *54* (16), 11169.

[3]	J. P. Perdew, K. Burke, M. Ernzerhof, *Phys. Rev. Lett.* **1996**, *77* (18), 3865.

[4]	G. Kresse, D. Joubert, *Phys. Rev. B* **1999**, *59* (3), 1758.

[5]	P. E. Blöchl, *Phys. Rev. B* **1994**, *50* (24), 17953.

[6]	L. Cheng, W. Li, Z. Chen, J. Ai, Z. Zhou, J. Liu, *Appl. Surf. Sci.* **2017**, *411*, 394.

[7]	Y. Xia, R. Tong, J. Zhang, M. Xu, G. Shao, H. Wang, Y. Dong, C.-A. Wang, *Nano-Micro Lett.* **2024**, *16* (1), 82.

[8]	D. Luo, X. Ma, P. Du, Z. Chen, Q. Lin, Y. Liu, B. Niu, X. He, X. Wang, *Angew. Chem., Int. Ed.* **2024**, *63* (28), 202401163.

[9]	Z. Zhu, H. Lu, D. Zhang, X. Jiang, C. Duan, Y. Qin, X. Yuan, Y. Jin, *J. Energy Storage* **2024**, *92*, 112211.

[10]	A. Naveed, T. Li, A. Ali, F. Ahmad, W. A. Qureshi, M. Su, X. Li, Y. Zhou, J.-C. Wu, Y. Liu, *Small* **2024**, *20* (40), 2401589.

[11]	Z. Jiao, X. Cai, X. Wang, Y. Li, Z. Bie, W. Song, *Adv. Energy Mater.* **2023**, *13* (48), 2302676.

[12]	Y. Li, P. Wu, W. Zhong, C. Xie, Y. Xie, Q. Zhang, D. Sun, Y. Tang, H. Wang, *Energy Environ. Sci.* **2021**, *14* (10), 5563.

[13]	W. Guo, L. Xu, Y. Su, L. Zhao, Y. Ding, Y. Zou, G. Zheng, T. Cheng, J. Sun, *Angew. Chem., Int. Ed.* **2025**, *64* (5), e202417125.

[14]	Z. Liu, R. Wang, Q. Ma, J. Wan, S. Zhang, L. Zhang, H. Li, Q. Luo, J. Wu, T. Zhou, J. Mao, L. Zhang, C. Zhang, Z. Guo, *Adv. Funct. Mater.* **2024**, *34* (5), 2214538.

[15]	Z. Yi, C. Luo, H. Wang, N. Ma, Y. Xie, J. Wu, Z. Lan, F. Yu, L. Que, L. Wang, *Adv. Mater.* **2025**, *38* (10), e19444.

[16]	C. Wang, X. Wang, H. Wang, C. Zheng, S. Tan, Y. Wang, G. Diao, Z. Jin, *Adv. Funct. Mater.* **2025**, *35* (41), 2424024.

[17]	B. Cai, Y. Sun, F. Yu, L. Wang, X. Yang, Y. Yang, X. Li, Y. Jiang, W. Lu, *Angew. Chem., Int. Ed.* **2026**, *0*, e21382.

[18]	C. Shen, Y. Zhang, X. Li, P. Guo, X. Zeng, K. Ni, R. Cao, Z. Wang, Z. Wang, L. Qin, *J. Mater. Chem. A* **2025**, *13* (3), 2174.

[19]	Y. Yang, Y. Li, Q. Zhu, B. Xu, *Adv. Funct. Mater.* **2024**, *34* (32), 2316371.

[20]	R. Luo, X. Zheng, T. Jiang, D. Shen, M. Wang, M. Ali, H. Liu, Z. Zhang, Y. Feng, S. Hazoor, P. Tong, W. Chen, *Adv. Funct. Mater.* **2025**, *15* (38), 2501658.
